# Andrographolide ameliorates OVA-induced lung injury in mice by suppressing ROS-mediated NF-κB signaling and NLRP3 inflammasome activation

**DOI:** 10.18632/oncotarget.12918

**Published:** 2016-10-26

**Authors:** Shuang Peng, Jian Gao, Wen Liu, Chunhong Jiang, Xiaoling Yang, Yang Sun, Wenjie Guo, Qiang Xu

**Affiliations:** ^1^ State Key Laboratory of Pharmaceutical Biotechnology, School of Life Sciences, Nanjing University, Nanjing, China; ^2^ State Key Laboratory of Innovative Nature Medicine and TCM Injections, Jiangxi Qingfeng Pharmaceutical Co., Ltd., Ganzhou, China

**Keywords:** andrographolide, asthma, NF-κB, NLRP3, ROS, Immunology and Microbiology Section, Immune response, Immunity

## Abstract

In this study, we attempted to explore the effect and possible mechanism of Andrographolide on OVA-induced asthma. OVA challenge induced significant airway inflammatory cell recruitment and lung histological alterations, which were ameliorated by Andrographolide. The protein levels of cytokines in bron-choalveolar fluid (BALF) and serum were reduced by Andrographolide administration as well as the mRNA levels in lung tissue. Mechanically, Andrographolide markedly hampered the activation of nuclear factor-κB (NF-κB) and NLRP3 inflammasome both *in vivo* and *vitro* thus decreased levels of TNF-α and IL-1β. Finally, we confirmed that ROS scavenging was responsible for Andrographolide's inactivation of NF-κB and NLRP3 inflammasome signaling. Our study here revealed the effect and possible mechanism of Andrographolide on asthma, which may represent a new therapeutic approach for treating this disease.

## INTRODUCTION

Lung injuries and its related chronic respiratory disorders which could be death threaten, are widespread healthy problems over the world [1]. Asthma, a respiratory disorder with lung injuries, is characterized by airway inflammation and bronchial hyper-responsiveness. It was estimated in 2012 that 18 million Americans had asthma [2]. Asthma may ultimately lead to lung function failure for unresolved inflammation-induced airway remodeling, which can barely be cured by current therapies. Commonly, asthma usually thought to be caused by the aberrant expansion of Th2 cytokines including IL-4, IL-5 as well as IL-13 [3, 4]. Indeed, studies have proposed that pattern recognition receptors (PRR) including TLR4 are necessary for Th2 responses upon pattern-associated molecular patterns (PAMPs) like LPS [5]. TLRs are the archetype transmembrane PRRs, which function through extracellular ligand recognition [6]. Besides the TLRs, the nucleotide-binding domain leucine-rich repeat-containing (NLR) family of PRRs for damage-associated molecular patterns (DAMPs) are also dispensable for regulation of innate immune response [7, 8]. Studies have proved that NLRP3, one of NLR family membranes, has a substantial impact on tissue inflammation including respiratory diseases [1, 9, 10]. The NLRP3 inflammasome complex, which is composed by NLRP3, ASC and CASP1, contributes to recognition of unique microbial and danger components. It also functions as a platform for CASP1 activation, which is responsible for IL-1β/IL-18 maturation [11]. NLRP3 inflammasome plays crucial role for host defense against infection. However, when excessive activated, it will lead to serious inflammatory conditions [12]. Thus target for NLRP3 inflammasome activation has been suggested as a useful strategy for inflammatory disease control.

Andrographolide, is a natural diterpenoid from *Andrographis paniculata*. It has been used in China for hundred years and now it has been officially approved as treatment mainly for respiratory infection and fever et al. Previously, we have reported that Andrographolide could alleviate LPS-induced sepsis and acute lung injury [13-15]. Here, we examined the anti-asthma effect of Andrographolide and provided the data which showed that Andrographolide significantly reduced NF-κB and NLRP3 inflammasome activation and finally alleviated the symptoms in OVA-treated mice.

## RESULTS

### Andrographolide inhibited infiltration of inflammatory cells and pathological changes of OVA-induced lung injury in mice

**Figure 1:**
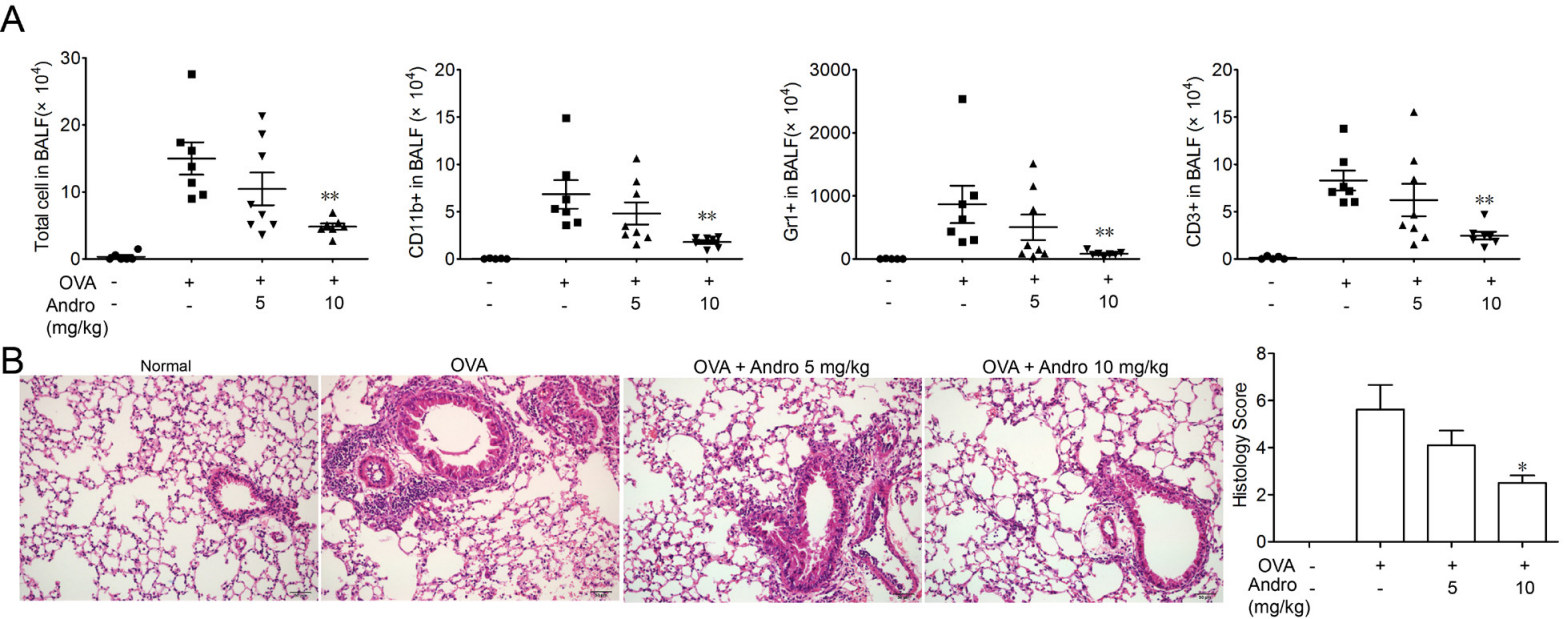
Andrographolide treatment ameliorated OVA-induced recruitment of inflammatory cells and lung injury in mice Mice were changed with OVA and treated with Andrographolide (5 and 10 mg/kg). **A.** BALF (bronchoalveolar lavage fluid) from each group was gathered and cells in BALF were counted. Then they were stained with CD3-FITC, CD11b-PE, CD11c-APC and analyzed by FACS. **B.** Lung tissue were fixed in 4% formalin and subjected to hematoxylin/eosin (H&E) staining. Values were shown as the means ± SEM of eight mice. *P < 0.05, **P < 0.01 *vs*. mice treated with OVA alone. Scale bar 50 μm.

In order to verify whether Andrographolide can improve asthma, we used a mouse model of OVA-induced lung inflammation to evaluate its effect. 24 h after the last OVA aerosol challenge, the mice were sacrificed and BALF was collected. Total and different kind of leukocyte cells in BALF were counted. OVA administration abviously increased total leukocyte cell counts as well as macrophage (CD11b+), lymphocyte (CD3+), and neutrophil (Gr1+) counts, as compared with normal mice (Figure 1A). Andrographolide administration significantly decreased the total leukocyte cell counts as well as macrophage, lymphocyte and neutrophil counts in BALF (Figure 1A).

Sections of lung tissues from OVA-challenged mice were stained with H&E and were showed in Figure 1B. OVA challenge caused significantly pathological alterations, such as infiltration of leukocyte cells, interstitial and intra-alveolar edema and patchy hemorrhage, formation of hyaline membrane, goblet cell hyperplasia and mucus hypersecretion. In contrary, Andrographolide administration markedly reduced these tissue injuries. The histology score also showed that Andrographolide dose-dependently alleviated lung tissue damage.

### Andrographolide inhibited levels of cytokines in BALF and serum

**Figure 2:**
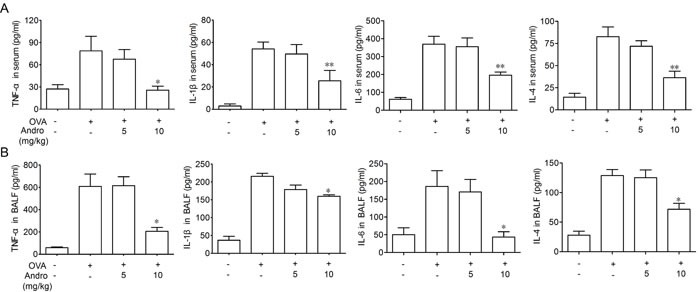
Andrographolide treatment suppressed OVA-induced cytokines elevation Cytokine levels in BALF supernatant **A.** and serum **B.** from each group were detected by ELISA. Values were shown as the means ± SEM of eight mice. *P < 0.05, **P < 0.01 *vs*. mice treated with OVA group.

After OVA inhaled-administration, leukocyte cells infiltration significantly increased and levels of inflammatory cytokines such as TNF-α, IL-6, IL-4 and IL-1β in BALF and serum were all elevated. However, these effects were attenuated by Andrographolide treatment. Data shown in Figure 2A, 2B indicated that Andrographolide significantly decreased the concentrations of cytokines both in BALF and serum.

### Andrographolide inhibited mRNA expressions of inflammatory cytokines

**Figure 3:**
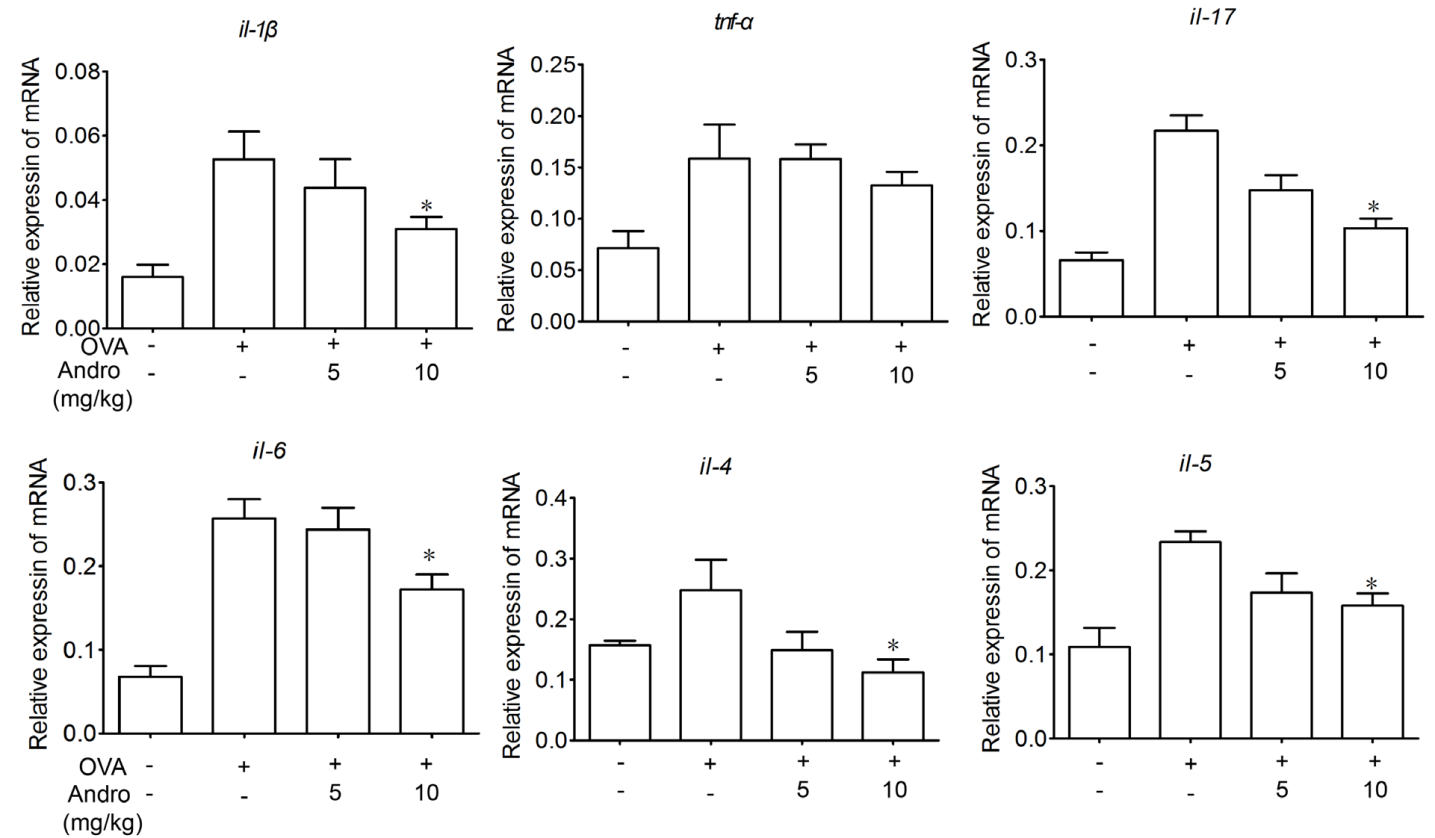
Andrographolide suppressed mRNA levels of inflammatory cytokines in lung of mice with OVA-induced asthma RNA of lung tissue from the mice was extracted. The mRNA expression of TNF-α, IL-1β, IL-17, IL-6, IL-4, IL-5 was examined by real-time PCR. Values were shown as the means ± SEM of eight mice. *P < 0.05, **P < 0.01 *vs*. mice treated with OVA alone.

As levels of TNF-α, IL-6, IL-4 as well as IL-1β in BALF and serum were suppressed by Andrographolide, we wonder whether it could also inhibit the the mRNA expressions of these cytokines in the injured lung. As we can see in Figure 3, the mRNA levels of IL-4, IL-5, IL-1β, IL-17A and IL-6 in homogenated lung protein from each group were apparently raised after OVA stimulation which has been significantly inhibited by Andrographolide treatment.

### Andrographolide reduced activation of NF-κB signaling

**Figure 4:**
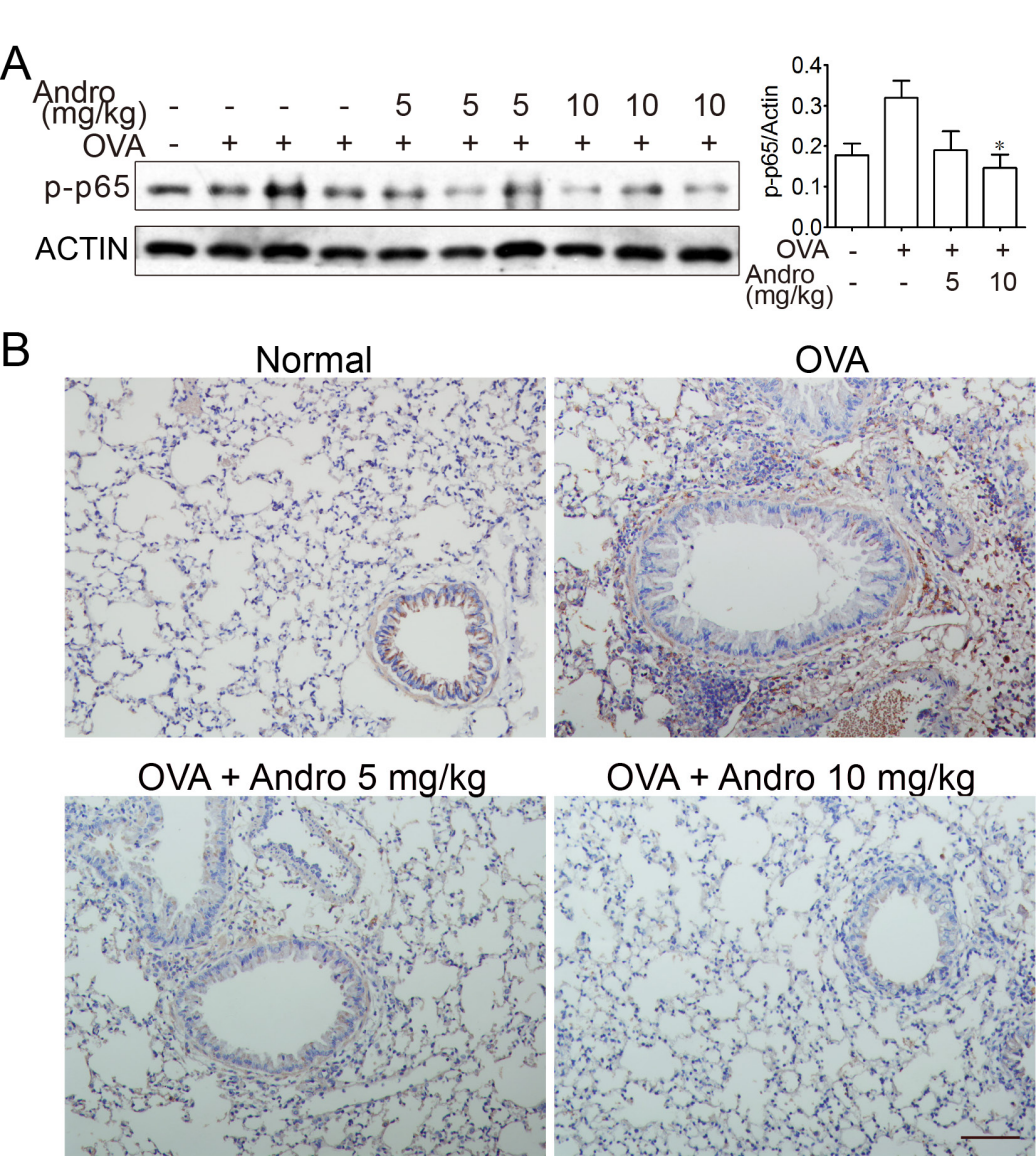
Andrographolide decreased activations of NF-κB in the lung of mice with OVA-induced asthma **A.** Lung tissue protein was extracted from the treated mice and protein level of p-p65 was examined by western blot. **B.** Paraffin-embedded lung tissue sections from vehicle- and Andrographolide-treated group were stained for p-p65. Scale bar 50 μm.

NF-κB plays key role during transcription of inflammation related genes. As shown in Figure 4A, activation of NF-κB signaling after OVA treatment was evidenced by elevated p65 phosphorylation level in the lungs from OVA-treated mice. Andrographolide treatment markedly reduced the phosphorylation of p65. Immunohistochemical stain revealed an increased level of p-p65 after OVA stimulation, while Andrographolide markedly decreased the p65 phosphorylation level (Figure 4B).

### Andrographolide reduced OVA-induced NLRP3 inflammasome activation in mice

**Figure 5:**
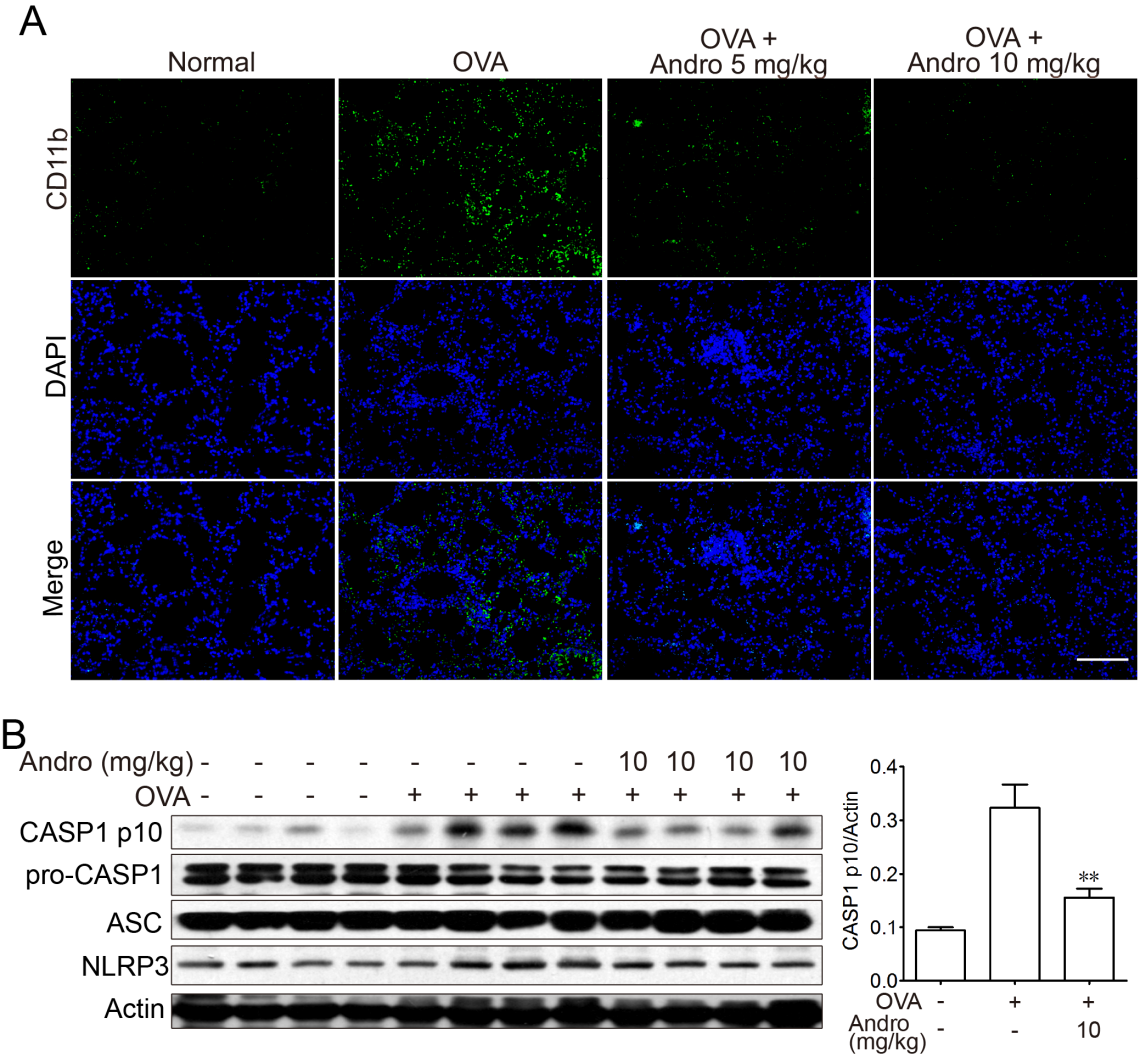
Andrographolide inhibited NLRP3 inflammasome activation in mice with OVA-induced asthma **A.** Lung tissue protein was extracted from the treated mice and protein levels of CASP1, NLRP3 and ASC were examined by western blot. **B.** Paraffin-embedded lung tissue sections each group were immunostained with DAPI (blue) and anti-CD11b-FITC (green) and observed by fluorescence microscopy, ×100. Scale bar 50 μm.

As shown in Figure 2, Andrographolide administration reduced IL-1β level *in vivo*. It has been documented that NLRP3 inflammasome which is responsible for IL-1β maturation may play crucial roles in OVA-induced asthma [1, 9]. To further investigate the mechanism of this reduced IL-1β production by Andrographolide, we examined macrophage infiltration in the lung tissues. Consistent with the local inflammation and other pathogenesis, an accumulation of large number of CD11b^+^ macrophages were observed in lung tissue samples from OVA-treated mice (Figure 5A). In contrast, Andrographolide treatment led to significant improvement in macrophage infiltration, as evidenced by diminished FITC-positive cells by immunofluorescent staining. In addition, CASP1 activation in lung tissue of mice from Andrographolide-treated groups was also reduced (Figure 5B). Our results showed that OVA-induced NLRP3 inflammasome activation in mice was suppressed by Andrographolide.

### Andrographolide inhibited NF-κB signaling *in vitro*

**Figure 6:**
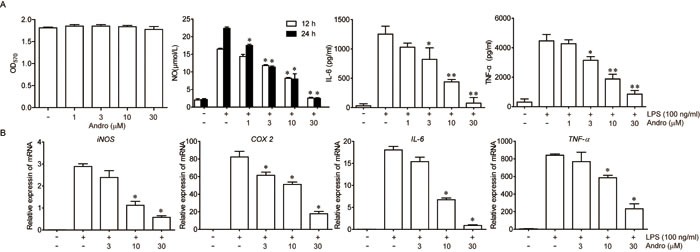
Andrographolide inhibited LPS-induced NO, IL-6 and TNF-**α** production in macrophages Raw264.7 cells were treated with various concentrations of Andrographolide in the absence or presence of LPS. **A.** The survival rate of 24 h was determined by MTT assay in the presence of Andrographolide. The levels of NO, IL-6 and TNF-α in the cell culture medium were determined 24 h after LPS stimulation as described in Methods. **B.** mRNA levels of iNOS, COX2, IL-6 and TNF-α in cells were examined 6 h after LPS stimulation by RT-PCR. Values were shown as the means ± SEM of three independent experiment. *P < 0.05, **P < 0.01 *vs*. LPS group.

Next, we aimed to explore the mechanism for the anti-inflammation effects of Andrographolide. Firstly, the effect of Andrographolide on NF-κB in macrophage *in vitro* was detected. As shown in Figure 6A, Andrographolide had no toxicity on Raw264.7 cells. Under LPS stimulation, content of NO, IL-6 or TNF-α in cell culture supernatant as well as mRNA levels of iNOS, COX2, IL-6 and TNF-α in cells were all elevated. Co-incubation with Andrographolide (1-30 μM) significantly decreased the levels of these cytokines (Figure 6A and 6B).

**Figure 7:**
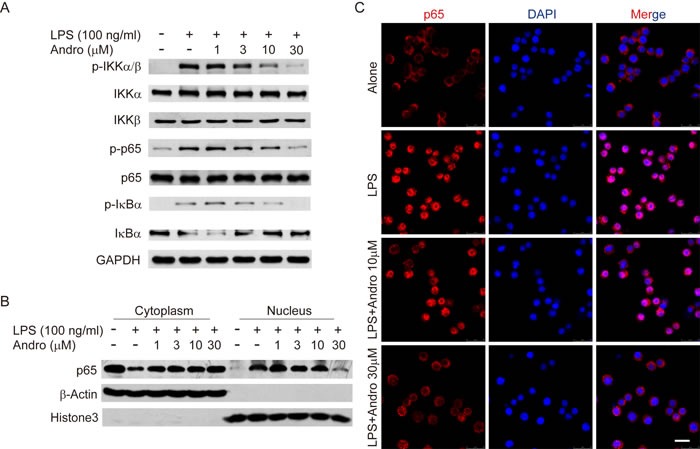
Andrographolide inhibits LPS-induced activation of NF-κB in RAW264.7 cells Cells were treated with various concentrations of Andrographolide in the absence or presence of LPS for 20 mins. **A.** The protein levels of phosphorylated and total IKKα, IKKβ, p65 and IκBα were determined by western blot. **B.** p65 levels in the cytosol and nucleus were assayed. β-actin and Histone3 were shown as loading controls. **C.** The subcellular localization of p65 was assayed by Immunofluorescence via confocal microscope. Nuclei were stained by DAPI. In all experiments, the representative data are shown of at least 3 independent experiments. Scale bar 20 μm.

The regulation effect of NF-κB during inflammation process and expression of iNOS or COX2 has been well illustrated [16, 17]. Thus, the effects of Andrographolide treatment on the activation of NF-κB stimulated by LPS in Raw264.7 cells were examined. As shown in Figure 7A, LPS-triggered phosphorylation of IKKα/β as well as the phosphorylation of IκBα were inhibited by Andrographolide while total IKKα or IKKβ level remains unchanged. Thereafter, the phosphorylation of p65 was also restrained by Andrographolide (Figure 7A). After phosphorylated triggered by LPS, p65 will translocate to nuclear, which was also decreased in the Andrographolide-treated cells proved by western blot of cytosol/nucleus contents and immunofluorescence staining (Figure 7B and 7C). Taken together, all these results proposed that Andrographolide inhibited NF-κB activation thereby reducing the secretion of cytokines.

### Andrographolide inhibited the OVA-induced activation of NLRP3 inflammasome *in vitro*

**Figure 8:**
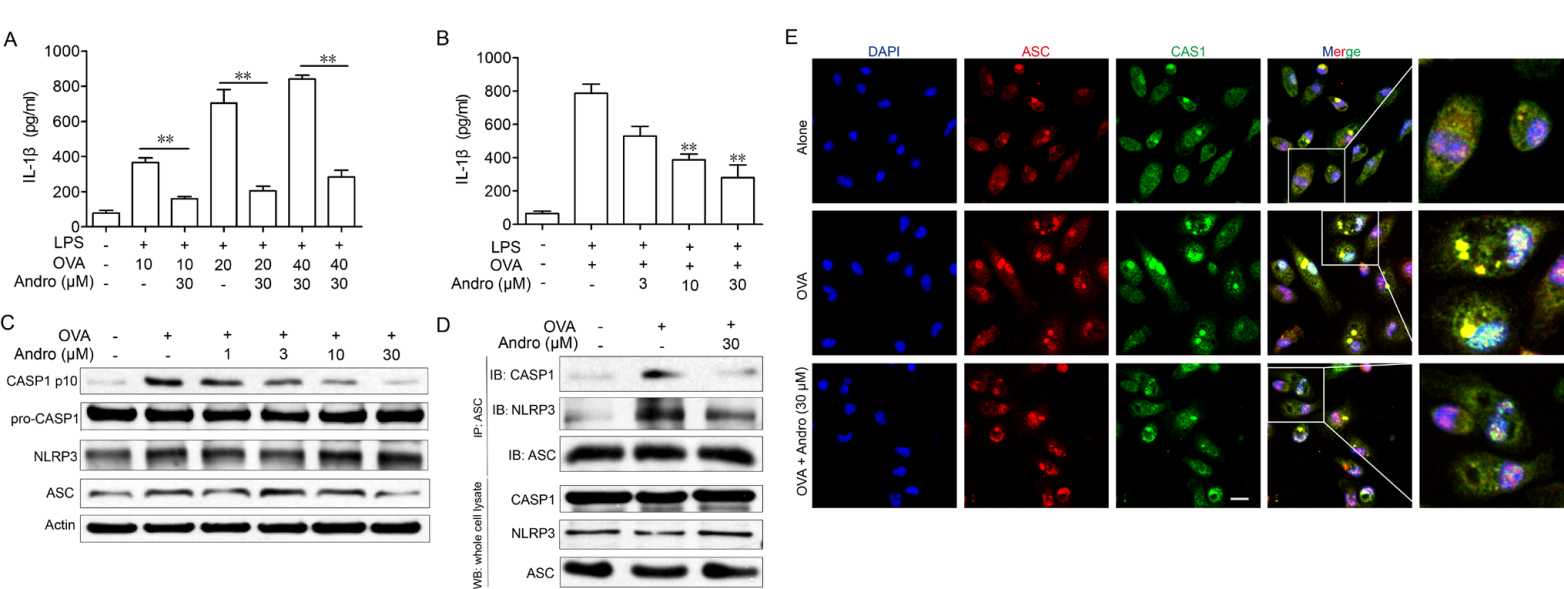
Andrographolide inhibited CASP1 activation and IL-1β maturation by inhibiting activation of the NLRP3 inflammasome **A.** LPS-primed BMDM cells were treated with Andrographolide (30 μM) with various OVA for 6 h, IL-1β levels in the supernatant were analyzed by ELISA. **B.** LPS-primed BMDM cells were treated with Andro (3, 10, 30 μM) with 40 μM OVA for 6 h, IL-1β levels in the supernatant were analyzed by ELISA. followed by 1 h incubation with 40 μM OVA. **C.** Protein levels of pro-CASP1, CASP1 p10, ASC and NRLP3 were determined by western blot. **D.** Proteins were isolated and immunoprecipitated with an antibody against ASC. **E.** BMDM cells were treated with Andro (30 μM) with 40 μM OVA for 6 h. Cells were analyzed by immunofluorescent cytochemistry (×100). All data shown are representative of three experiments. *P < 0.05, **P < 0.01 *vs*. LPS+OVA-treated group. Scale bar 20 μm.

Besides the NF-κB signaling, recent evidence indicates that IL-1β was transcript as an inactive form (pro-IL-1β) which need to be cleaved by activated CASP1 to release the mature form. The data got from cell line studies showed that Andrographolide could inhibit OVA-activated NLRP3 inflammasome. Logically, we next aimed to examine the detail mechanism *in vitro*. As shown in Figure 8A and 8B, LPS plus OVA stimulation exhibited an obvious raise of IL-1β production in BMDM while Andrographolide dose-dependently decreased it. Examination of CASP1 expression showed that Andrographolide inhibited OVA-induced CASP1 cleavage *in vitro* (Figure 8C), which is in line with the results *in vivo* (Figure 5B). Upon OVA stimulation, NLRP3 recruited ASC and ASC in turn recruited pro-CASP1, which resulted in autocatalysis and activation of CASP1, a key event in NLRP3 inflammasome activation. Immunoprecipitation (IP) showed that Andrographolide treatment inhibited the association of ASC and pro-CASP1 or NLRP3 (Figure 8D). Immunofluroscence analysis (Figure 8E) revealed that Andrographolide markedly interrupted the co-localization of ASC and pro-CASP1, which subsequently inhibited cleavage of pro-CASP1. Collectively, these observations suggest that Andrographolide can inhibit NLRP3 inflammasome-mediated CASP1 activation by decreasing the assembly of NLRP3/ASC/CASP1 complex.

### Andrographolide inhibited ROS generation

Next, we sought to determine how Andrographolide inhibited the signaling of NF-κB and NLRP3 inflammasome. Under LPS stimulation, the TLR4-mediated reactive oxygen species (ROS) generation was dispensable for NF-κB activation and NLRP3 inflammasome complex formation as well as the following production of the inflammatory cytokines including TNF-α and IL-1β [18, 19].

**Figure 9:**
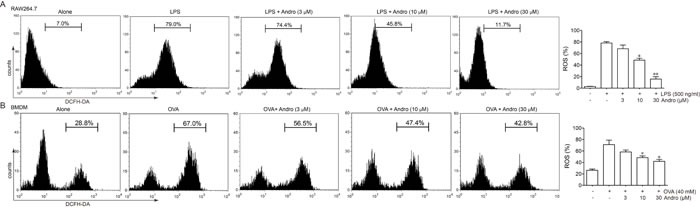
Andrographolide inhibited LPS and OVA triggered ROS generation **A.** Raw264.7 Cells were treated with various concentrations of Andrographolide in the absence or presence of 100 ng/ml LPS for 6 h. **B.** BMDM were treated with various concentrations of Andrographolide in the absence or presence of 40 μM OVA for 6 h. ROS level was examined by DCFH stain. *P < 0.05, **P < 0.01 *vs*. LPS or OVA-treated group.

Thus, we wonder whether Andrographolide could reduce ROS generation under LPS or OVA stimulation. As showed in Figure 9A and 9B, either LPS or OVA stimulation caused apparent ROS production in cells, while 10 and 30 μM Andrographolide treatment reduced its production.

## DISCUSSION

Asthma is a chronic inflammatory disease of the respiratory airways with an increasing prevalence. Risk factors such as changing patterns of air, air pollution, unplanned urbanization, packed cohabitation, etc. promote Asthma development [20, 21]. Asthma can impair the quality of daily life while may even be life-threaten thus needs to be carefully managed. Many drugs including corticosteroids and the TNF-α antibody infliximab were used for Asthma therapy via reducing macrophage-mediated inflammation. However, serious side effects such as steroid dependence and serious infections would be caused for long time use [22, 23]. Therefore, it is necessary for seek of safe and effective new agents. Our study here aimed to examine the effects and mechanisms of Andrographolide on OVA-induced asthma *in vivo* and *vitro*. Our data showed that Andrographolide inhibited OVA-induced lung histopathological changes, inflammatory cell infiltration, the levels of cytokines in BALF and serum, providing the possibility that Andrographolide could be used against asthma.

It is known the transcription factor NF-κB activated by LPS, mediates the expression of pro-inflammatory cytokines, playing an important role in many inflammatory diseases [24-26]. It has been proved by others that Andrographolide can bind to p50 and inhibit NF-κB activation [27]. Our research here confirmed that Andrographolide treatment could reduce the phosphorylation of IKKα/β, IκBα and NF-κB which suggest that Andrographolide may work on the very upstream of LPS-TLR4-NF-κB pathway.

Besides NF-κB signaling, a key role for the NLRP3 inflammasome-mediated cytokine maturation in the pathology of allergic airway disease has also been proposed. CASP1 activation has been demonstrated in mouse model and also in our experiments [28]. NLRP3, ASC, CASP1 or IL-1β knockout mice showed significantly decreased symptoms in response to OVA [29, 30]. Notably, NLRP3 expression in CD4+ T cells can even specifically and positively regulate TH2 cells program [31].

As proved by our data, Andrographolide could both suppress LPS-induced NF-κB and OVA-induced NLRP3 activation and we wondered whether these two pathways have common characteristics which were targeted by Andrographolide.

Reactive oxygen species (ROS), as a pivotal intracellular messengers in response to various stimulations are known to play a prominent role in many physiology or pathogenesis process [32, 33]scavenger ROS seems to be a general mediators for NLRP3 activation [38]. Different from LPS-induced ROS generation from, ROS mainly derived from mitochondria by NLRP3 stimulators [39, 40]. However, Andrographolide could eliminate ROS generated from either situation.

Above all, our data here showed that Andrographolide can inhibit OVA-induced asthma with a possible mechanism of ROS elimination, thus negatively regulate NF-κB and NLRP3 inflammasome activation and attenuate lung inflammation/injury in mice. These results provide the evidence for the possible clinical application of Andrographolide for asthma or inflammatory airway diseases.

**Figure 10:**
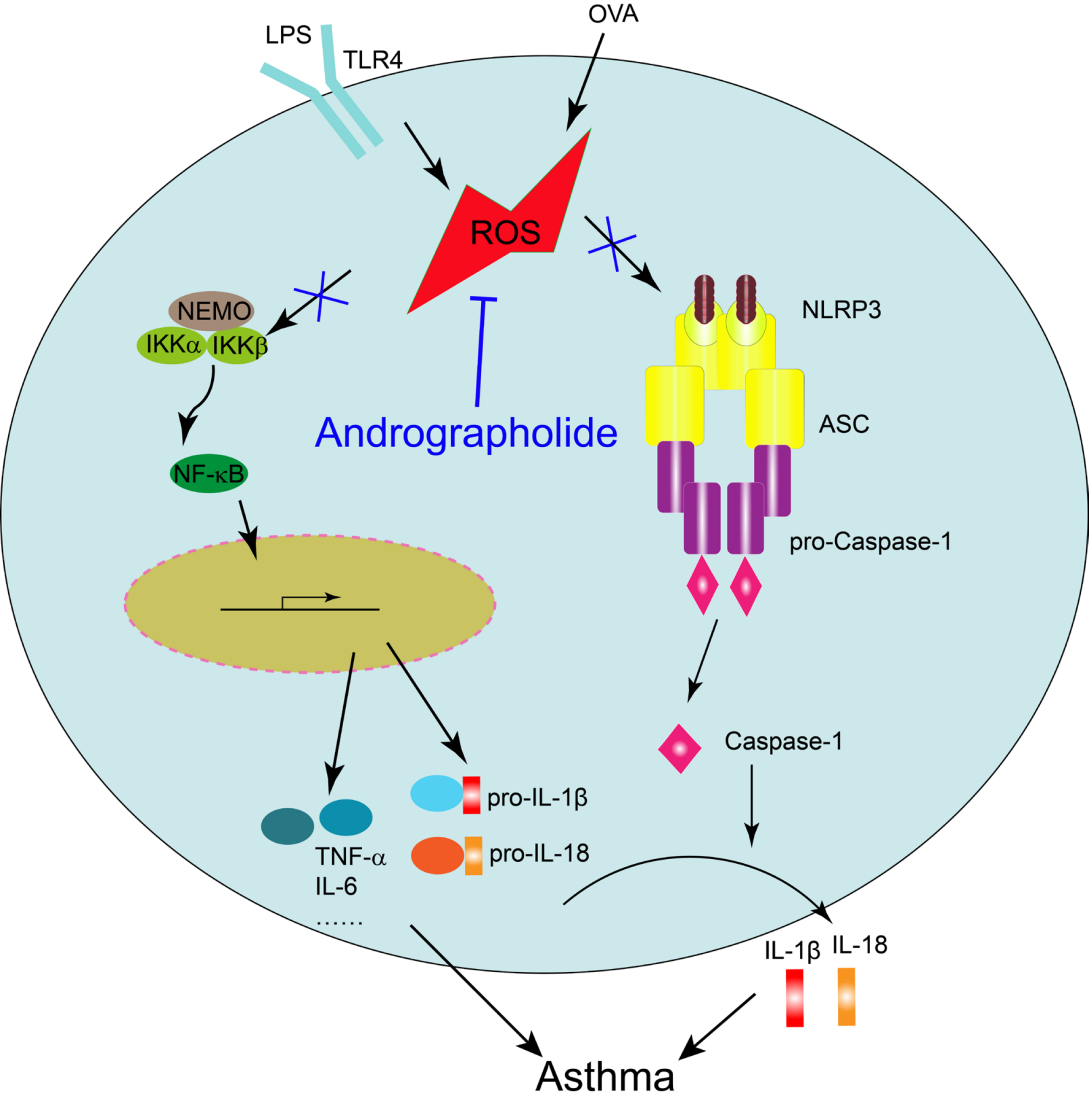
Illustration for the mechanism underlying Andrographolide for improvement of OVA-induced lung inflammation in mice Andrographolide ameliorates OVA-induced lung injury in mice by suppressing ROS-mediated NF-κB signaling and NLRP3 inflammasome activation.

## MATERIALS AND METHODS

### Animals

Female C57/BL6 mice, 6-8 weeks of age, were purchased from Model Animal Research Center of Nanjing University (Nanjing, China). Mice were maintained in an animal facility under standard laboratory conditions for 1 week prior to experiments, and provided water and standard chow. Animal welfare and experimental procedures were carried out in accordance with the Guide for the Care and Use of Laboratory Animals (Ministry of Science and Technology of China, 2006). All efforts were made to reduce the number of animals used and to minimize animals' suffering.

### Agents

OVA, LPS and Andrographolide were purchased from Sigma-Aldrich (St. Louis, MO). Water-soluble Andrographolide sulfonate (Xi-Yan-Ping Injection) was provided by Jiangxi Qingfeng Pharmaceutical Co., Ltd. ELISA kits for TNF-α, IL-6, IL-4 and IL-1β were purchased from Dakewei (Beijing, China). Anti-phosphorylation of p65 and anti-p-p65 were purchased from Cell Signaling Technology (Beverly, MA). Anti-NLRP3 and anti-CASP1 (3345-1) were purchased from Epitomics. Anti-ASC and anti-Actin (sc-1616) were purchased from Santa Cruz Biotechnology (Santa Cruz, CA). Anti-mouse CD3-FITC, CD11b-PE and CD11c-APC antibodies were bought from eBioscience. GTVisin™ anti-mouse/anti-rabbit immunohistochemical analysis KIT was purchased from Gene Company (Shanghai, China). All other chemicals were obtained from Sigma-Aldrich (St. Louis, MO).

### Induction of asthma by OVA

C57/BL6 mice were i.p administered with 20 μg OVA in aluminum potassium sulfate or aluminum potassium sulfate (control mice) on days 0, 7 and challenged consecutively by aerosol inhalation on days 14-28 (20 mg/ml OVA in PBS, 30 min/day). Mice were randomly divided into five groups (*n* = 8 per group): normal group, OVA-treated group (model) and OVA+Andro treated groups. Andrographolide sulfonate (5, 10 mg/kg) dissolved in saline were given to mice once per day (i.p) from day 10-14. 3 days after the last OVA challenge, mice were killed and BALF, serum and tissue samples were collected and assessed for allergic airway development. For BALF collection, mice were perfused with ice cold PBS. The lungs were lavaged with 300 μl saline, and the resultant BALF was centrifuged to separate the cellular components from the supernatants. Total BALF cell number was counted and the BALF cells were stained with anti-mouse CD3-FITC, CD11b-PE, CD11c-APC antibodies and composition was evaluated by FASC analysis.

### Histological analysis

To examine the histological changes, lungs from animals in each group were taken and fixed by 10% formalin, embedded in paraffin, and then sectioned to reveal the maximum longitudinal view of the main intrapulmonary bronchus of the left lung lobe. Histopathologic study was made using hematoxylin & eosin (H&E)-stained lung sections. Alveolar congestion, haemorrhage, infiltration or aggregation of inflammatory cells in airspaces or vessel walls, and the thickness of the alveolar walls were assessed.

### Cytokine analysis by ELISA

Serum was collected from blood of mice by centrifuge at 3500 *g* for 15 min. Serum cytokine levels were measured by specific ELISA kits from Dakawe (Beijing, China).

### Real-time PCR

Real-time PCR was performed as follows as previously reported [41]: RNA samples from lung tissues of mice in each group were extracted and reversed to cDNA and subjected to quantitative PCR, which was performed with the BioRad CFX96 Touch™ Real-Time PCR Detection System (BioRad, USA) using iQ™ SYBR^®^ Green Supermix, and threshold cycle numbers were obtained using BioRad CFX Manager Software. The program for amplification was 1 cycle of 95°C for 2 min followed by 40 cycles of 95°C for 10 s, 60°C for 30 s, and 95°C for 10 s. The primer sequences used in this study were as follows:

*tnf-α* forward 5’-CGAGTGACAAGCCTGTAGCCC-3’;

*tnf-α* reverse 5’-GTCTTTGAGATCCATGCCGTTG-3;

*il-1β* forward 5’-CTTCAGGCAGGCAGTATCACTC-3’;

*il-1β* reverse 5’-TGCAGTTGTCTAATGGGAACGT-3’;

*il-6* forward 5’-ACAACCACGGCCTTCCCTAC-3’;

*il-6* reverse 5’-TCTCATTTCCACGATTTCCCAG-3’;

*il-4* forward 5’-GTCTGCTGTGGCATATTCTG-3’;

*il-4* reverse 5’-GGCATTTCTCATTCAGATTC-3’;

*il-5* forward 5’-GGCTACACAGAGAAACCCTGT-3’;

*il-5* reverse 5’-CATGCATACACAGGTAGTTCA-3’;

*Il17a* forward 5’-TCGAGAAGATGCTGGTGGGT-3’;

*Il17a* reverse 5’-CTCTGTTTAGGCTGCCTGGC-3’;

*β-actin* forward 5’-TGCTGTCCCTGTATGCCTCT-3’;

*β-actin* reverse 5’-TTTGATGTCACGCACGATTT-3’.

### Western blot

Protein from lung tissue was extracted in lysis buffer (30 mmol/L Tris, pH 7.5, 150 mmol/L sodium chloride, 1 mmol/L phenylmethylsulfonyl fluoride, 1 mmol/L sodium orthovanadate, 1% Nonidet P-40, 10% glycerol, and phosphatase and protease inhibitors). The protein content of the supernatant was determined by BCA protein assay Kit (Pierce, Rochford, IL). The proteins were then separated by SDS-PAGE and electrophoretically transferred onto polyvinylidene fluoride membranes. The membrane was blocked with 5% nonfat milk for 1 h at room temperature. The blocked membrane was incubated with the indicated primary antibodies overnight at 4 °C, and then incubated with HRP-coupled secondary antibody. Detection was performed using a LumiGLO chemiluminescent substrate system (KPL, Guildford, UK).

### Cell culture

Murine RAW264.7 macrophages, obtained from the American Type Culture Collection (Rockville, MD), were cultured in DMEM (Invitrogen Corp, Carlsbad, CA) containing 5% fetal bovine serum (GIBCO, Grand Island, NY), 100 U/ml penicillin, and 100 mg/ml streptomycin in 5% CO_2_ at 37°C. Bone marrow derived macrophage (BMDM) cells were isolated according to the following procedures. Bone marrow cells were isolated from C57/BL6 mice and cultured with DMEM supplemented with 10% fetal bovine serum and 20 ng/ml GM-CSF (Peprotech, Rock Hill, NJ). Culture fluid was exchanged to fresh culture medium every 3 days. Under these conditions, adherent macrophages were obtained within 7 to 8 days. Cells were harvested and seeded on 24-well plates. After culturing for 6 h without GM-CSF, the cells were used for the experiments as bone marrow derived macrophages [42].

### NLRP3 inflammasome activation model *in vitro*

NLRP3 inflammasome activation model*in vitro* was established as follows: BMDM cells were pretreated with 100 ng/ml LPS for 3 hours and then incubated with various concentrations of OVA (10, 20, 40 μM) for 6 h, IL-1β levels in the supernatant were analyzed by ELISA.

### Immunohistochemical analysis

Immunohistochemical analysis was performed on paraffin-embedded lung tissue sections (5 μm) were done as described previously [41].

### Coimmunoprecipitation assay

Proteins from cells were incubated with 1 μg of appropriate antibody and precipitated with protein A/G-agarose beads (Santa Cruz Biotechnology, Santa Cruz, CA). The immunoprecipitated proteins were separated by SDS-PAGE and western blot was performed with the indicated antibodies.

### Measurement of intracellular ROS

Cells were cultured in a 6-well plate and treated with various concentrations of Andrographolide in the presence of LPS or OVA for 6 h. Then the cells were harvested and incubated with 2,7-dichlorofluorescein diacetate (DCFH-DA) at 37°C for 20 min and washed twice with cold PBS. DCF fluorescence distribution was detected by flow cytrometry on a FACScan (Becton Dickinson) at an excitation wavelength of 488 nm and an emission wavelength of 525 nm.

### Statistical analysis

Data were expressed as mean ± SEM. ANOVA with post hoc two comparisons was used to evaluate the differences between various experimental and control groups. *P* values less than 0.05 were considered significant.
